# MiR-483 induces senescence of human adipose-derived mesenchymal stem cells through *IGF1* inhibition

**DOI:** 10.18632/aging.103818

**Published:** 2020-08-15

**Authors:** Junyan Shen, Xiaoqi Zhu, Hailiang Liu

**Affiliations:** 1 Institute for Regenerative Medicine, Shanghai East Hospital, Tongji University School of Medicine, Shanghai 200123, China

**Keywords:** mesenchymal stem cell, senescence, adipogenesis

## Abstract

Human adipose-derived mesenchymal stem cells (hADSCs) are an ideal source of seed cells for regenerative applications and tissue engineering. However, long-term *in vitro* culture of hADSCs reduces their quantity and quality, which lessens their value in research and clinical applications. The molecular mechanisms underlying this biological process are poorly defined. Recently identified microRNAs (miRNAs) have emerged as critical modulators of cellular senescence. In this study, we examined the changes in hADSCs undergoing senescence. Significant miR-483-3p upregulation was noted during *in vitro* passaging of hADSCs, which correlated with the adipogenic differentiation and cellular senescence. Knockdown of miR-483-3p retarded the adipogenic differentiation potential of hADSCs and reduced cellular senescence. Dual-luciferase reporter assays identified insulin-like growth factor-1 (*IGF1*) as the target gene of miR-483-3p. *IGF1* inhibition confirmed its inhibitory effects on replicative senescence in hADSCs. In conclusion, our study revealed essential regulatory roles of miR-483-3p in the adipogenesis and aging of hADSCs mediated by targeting *IGF1*.

## INTRODUCTION

Mesenchymal stem cells (MSCs) are a type of stem cells that can be isolated from adipose tissues, bone marrow, and umbilical cord blood [1]. Recent studies have shown that MSCs can be used as pluripotent stem cells to repair damaged tissues, produce biologically active molecules to promote tissue regeneration and regulate the maturation and function of innate and adaptive immune effector cells [2, 3]. These features make MSCs ideal candidates for cell-based therapy of many diseases. Human adipose-derived mesenchymal stem cells (hADSCs) can easily be isolated and have strong differentiation and proliferation abilities *in vitro*, and have thus become the ultimate source of seed cells for regenerative applications and tissue engineering. However, long-term culture of MSCs results in altered cell morphology, slower proliferation rates and variations in differentiation potential [4, 5]. There is substantial evidence that the aging of MSCs contributes to age-related diseases and aging, and that replicative senescence damages the regenerative potential of MSCs [6]. It is crucial to understand the mechanisms that lead to replicative senescence in MSCs to evaluate potential approaches to maintaining their regenerative function and thereby produce more robust regenerative cells for cellular therapy.

MicroRNAs (miRNAs) are endogenous single-stranded RNAs composed of 19–25 nucleotides and regulate the expression of their complementary messenger RNAs (mRNAs) [7, 8]. Regulation by miRNAs leads to mRNA destruction or translational repression of target genes [9]. Previous studies have demonstrated that miRNAs play essential roles in various cellular and biological processes, including cell apoptosis, cell proliferation, cancer, and metabolic diseases [10–12].

In recent studies, miRNAs have emerged as modulators of the expression of genes associated with cellular senescence [13]. MiR-483 gene family is first discovered as part of intron 2 of insulin-like growth factor-2 (*IGF2*) [14]. MiR-483 suppresses chondrogenic differentiation of hMSCs [15]. However, the potential effects of miR-483 on the replicative senescence of hADSCs are obscure.

In this study, we investigated the following changes in hADSCs undergoing senescence: increased ability of adipogenic differentiation, shorted telomere length, enhanced activity of the acidic senescence-associated β-galactosidase (SA-β-gal), and increased expression of p16, p21, and p53. We demonstrated that knockdown of miR-483-3p inhibited aging-related adipogenic differentiation of hADSCs. Insulin-like growth factor-1 (*IGF1*) was identified as a target gene of miR-483-3p. We also discovered that miR-483-3p expression was markedly increased during senescence of hADSCs cultured *in vitro*. Ultimately, we identified miR-483-3p as a novel, direct molecular regulator involved in the replicative senescence of hADSCs.

## RESULTS

### Characterization and verification of senescent MSCs

The senescence phenotypes of hADSCs grown *in vitro* were assessed by isolating hADSCs from two different donors and subjecting to long-term proliferation under the same culture conditions. After passage 9 (P9), hADSCs gradually increased in size, and acquired a flat and irregular shape, which is the typical morphology of senescent cells (Supplementary Figure 1A, 1B). Flow cytometry analysis showed that hADSCs from early (P9) and late (P16) passages were both positive for the MSCs surface markers CD73, CD90, and CD105, but negative for CD34, CD45, CD11b, CD19 and HLA-DR (Supplementary Figure 1C, 1D).

**Figure 1:**
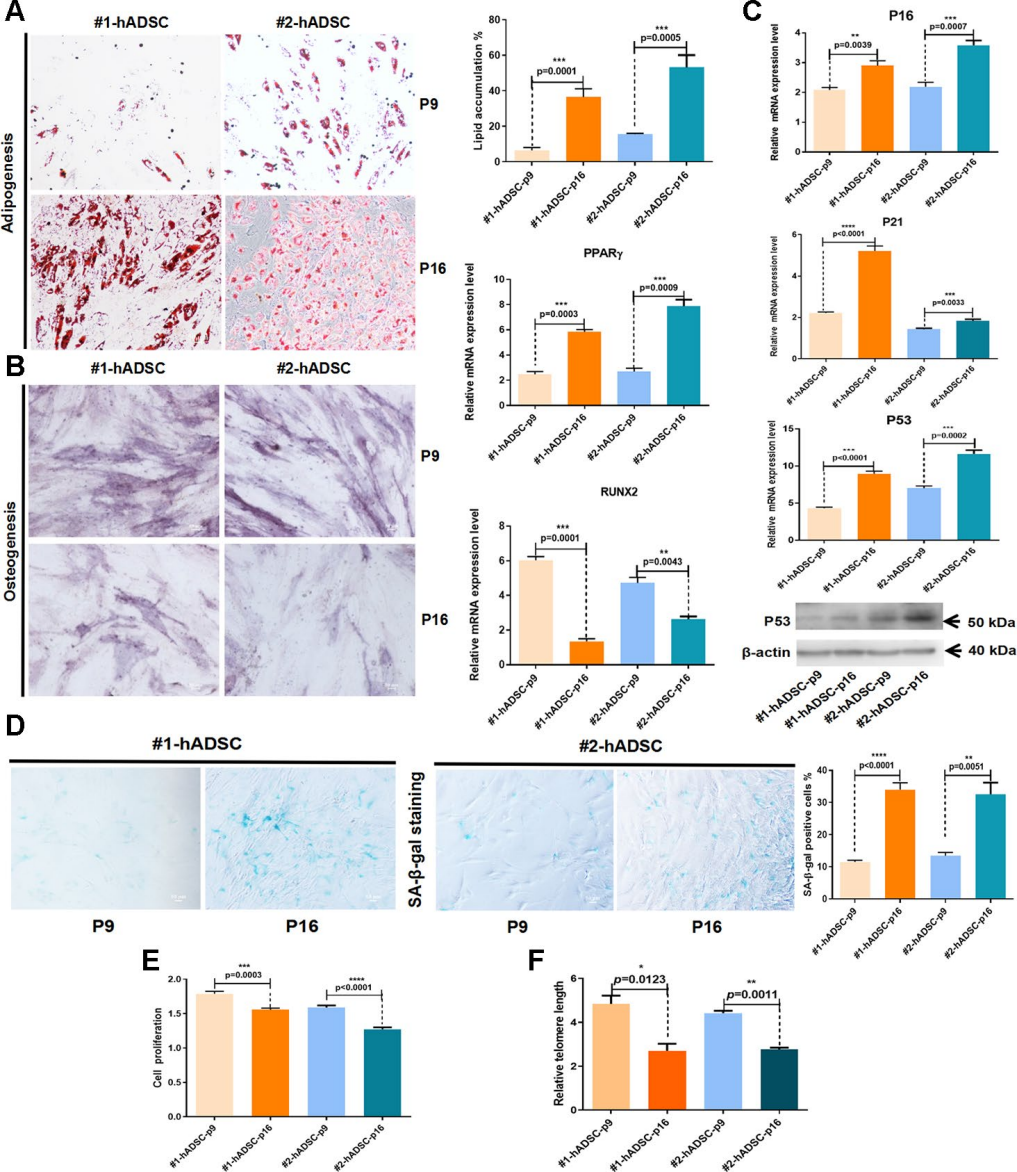
**Characterization and verification of senescent MSCs from human adipose tissues from two different donors (#1 and #2).** (**A**) Oil Red O staining of hADSCs at P9 and P16 (10×; scale: 50 μm); MRNA expression of PPARγ was evaluated by RT-qPCR. (**B**) Alkaline phosphatase staining of hADSCs at P9 and P16 (10×; scale: 50 μm); MRNA expression of RUNX2 was evaluated by RT-qPCR. (**C**) MRNA expression levels of p16, p21, and p53 were evaluated by RT-qPCR; western blot analysis of p53. (**D**) SA-β-gal staining of hADSCs at P9 and P16 (10×; scale: 50 μm). (**E**) The proliferation of hADSCs at P9 and P16 was detected by CCK-8 assay. (**F**) Telomere length was demonstrated by qPCR.

Another key criterion for the identification of MSCs is their potential to differentiate into osteoblasts and adipocytes. Here, we analyzed the effect of replicative senescence on the differentiation potential of hADSCs. Our findings revealed that hADSCs undergoing long-term culture had an increased adipocyte differentiation ability but a reduced osteocyte differentiation ability (Figure 1A, 1B).

Furthermore, the expression of three cell senescence-related molecular markers (p16, p21, and p53) were significantly increased from P9 to P16 (Figure 1C). Additionally, the number of cells that positively stained with SA-β-gal was significantly increased in P16 (Figure 1D). Cell counting kit-8 (CCK-8) assay results showed that the proliferation of hADSCs was significantly downregulated in P16 (Figure 1E). Changes in telomere length were evaluated using quantitative PCR (qPCR) [16], which revealed that telomere shortening occurred during the aging of hADSCs *in vitro* (Figure 1F). These data generally indicated that hADSCs exhibit a constant increase in senescence with successive passaging in culture.

### MiRNAs expression in the adipogenic differentiation of hADSCs

**Figure 2:**
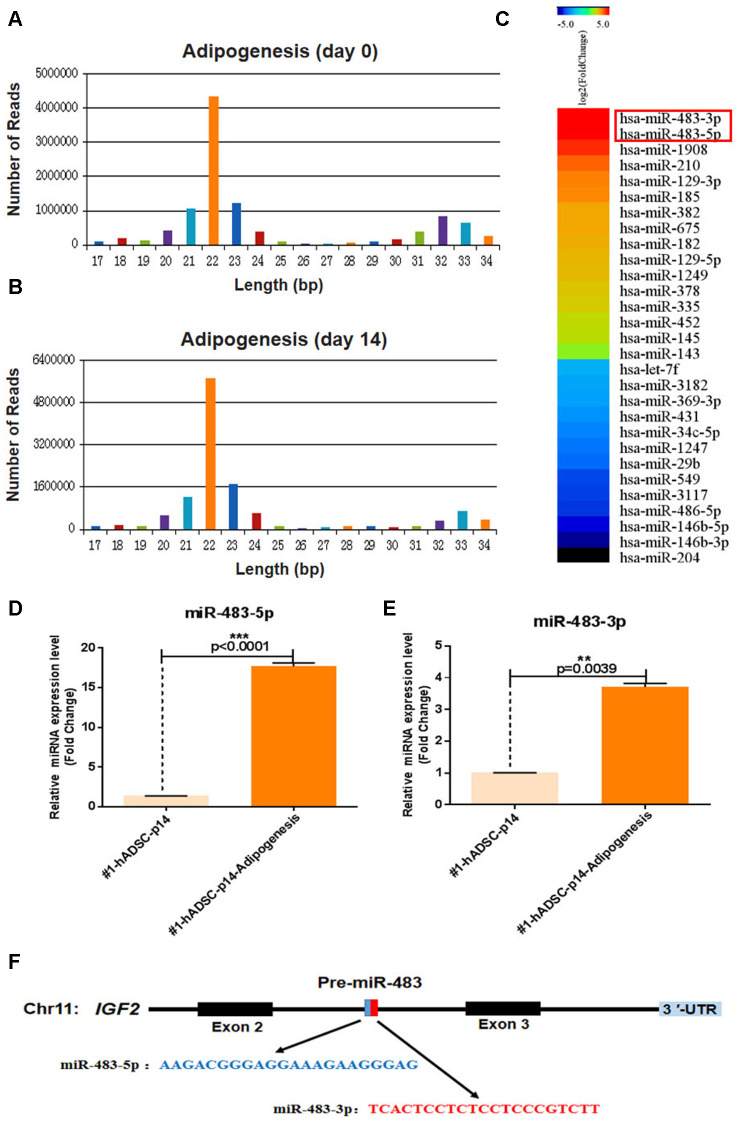
**MiR-483 expression during the adipogenic differentiation of hADSCs.** (**A**, **B**) Small-RNA sequencing was performed to profile miRNAs with variant expression during adipogenic differentiation of hADSCs. Data are expressed as the number of reads and (**C**) differentially expressed miRNA genes. (**D**, **E**) The relative expression of miR-483-5p and miR-483-3p was validated by RT-qPCR. (**F**) The location and classification of miR-483.

Previous studies have revealed that osteoblasts and adipocytes share a common mesenchymal ancestor and that the distinct processes of osteogenic and adipogenic differentiation from MSCs are competitive [17]. We found that adipogenic differentiation of hADSCs increased during cellular senescence which was consistent with previous findings [18]. Comparative sequencing of small RNA using adipogenic differentiation samples from day 0 (undifferentiated) and day 14 were performed to identify any putative miRNAs that simultaneously affect adipogenic differentiation and replicative senescence in hADSCs (Figure 2A, 2B).

Apart from miR-1908, which has been reported to be highly expressed during adipogenic differentiation of hADSCs [19], we found that miR-483-3p and miR-483-5p expression were also significantly increased during adipogenesis (Figure 2C). To further verify these results, miR-483-5p and miR-483-3p expression were analyzed using RT-qPCR (Figure 2D, 2E). As pre-miR-483 is located in intron 2 of the *IGF2* transcript and encodes two mature miRNAs (miR-483-3p/miR-483-5p) (Figure 2F), and *IGF2* modulates the differentiation of stem cells [20, 21], we also measured the *IGF2* expression using RT-qPCR. Consistently, *IGF2* and miR-483 displayed similar expression pattern in adipogenic differentiated hADSCs that were isolated from two different donors and cultured under the same conditions (Supplementary Figure 2). Therefore, we speculated that miR-483 plays a vital role in the differentiation of hADSCs.

### MiR-483 promotes adipogenesis of hADSCs *in vitro*

**Figure 3:**
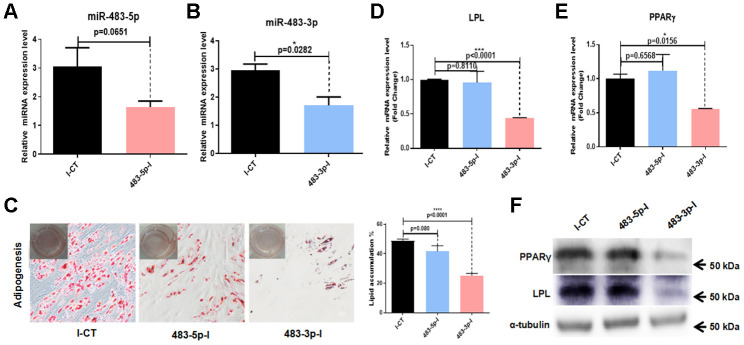
**Effects of miR-483 on adipogenic differentiation of hADSCs.** (**A**, **B**) The efficiency of miR-483-5p and miR-483-3p inhibition was assayed by RT-qPCR. (**C**) The effect of inhibition of miR-483-3p and miR-483-5p on adipogenic differentiation of hADSCs was evaluated on day 14 using Oil Red O staining (10×; scale: 50μm). (**D**–**F**) RT-qPCR and western blot analysis of LPL and PPARγ expression levels. I-CT: negative control inhibitor; 483-5p-I: inhibitor of miR-483-5p; 483-3p-I: inhibitor of miR-483-3p.

To further investigate the role of miR-483 in the differentiation of hADSCs, we transfected hADSCs with 483-3p-I (an inhibitor of miR-483-3p), 483-5p-I (an inhibitor of miR-483-5p), or negative control inhibitor (I-CT). The transfection efficiency was quantified at 48 hours after transfection by RT-qPCR. The success of the inhibition was demonstrated by low miR-483-5p and miR-483-3p expression (Figure 3A, 3B). We induced adipogenic differentiation of hADSCs transfected with 483-5p-I, 483-3p-I, or I-CT, then measured the adipogenic induction efficacy. It was found that inhibition of miR-483-5p and miR-483-3p in hADSCs decreased the number of lipid droplets (Figure 3C). To confirm these results, we investigated the expression of two primary adipogenic transcription factors, lipoprotein lipase (*LPL*) and peroxisome proliferator-activated receptor-gamma (*PPARγ*), by RT-qPCR (Figure 3D, 3E) and western blot analysis (Figure 3F). Compared with the control group, the mRNA and protein levels of LPL and PPARγ were significantly decreased in the 483-3p-I group, but not in the 483-5p-I group. Because miR-483-3p had the stronger effect of the two miRNAs, we selected it for further characterization.

### MiR-483-3p directly targets the 3′UTR of *IGF1* mRNA

**Figure 4:**
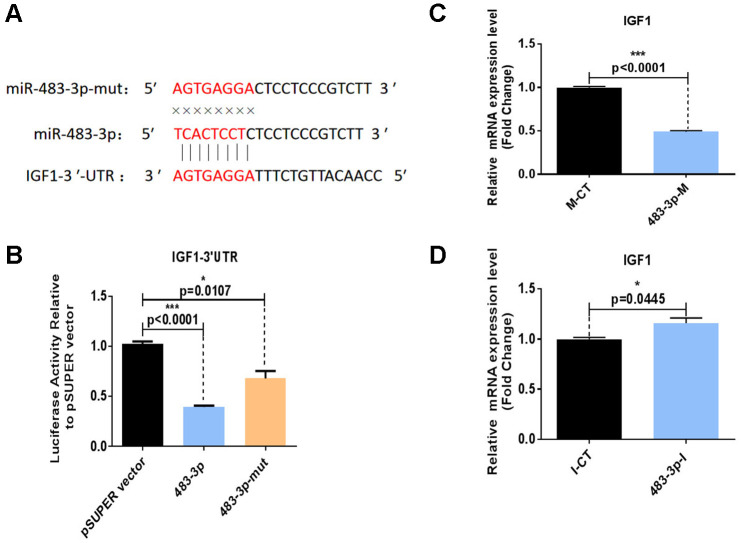
**Identification of *IGF1* as a direct target of miR-483-3p.** (**A**) Putative miR-483-3p target sites in *IGF1* and design of mutation sites. (**B**) Activity of the luciferase reporter gene in HEK-293T cells co-transfected with a luciferase reporter plasmid containing *IGF1* 3′ UTRs and wild-type pSUPER-miR-483-3p, mutant pSUPER-miR-483-3p, or empty pSUPER vector. Luciferase activity was assayed 32 hours after transfection. (**C**, **D**) RT-qPCR analysis of *IGF1* mRNA levels in hADSCs transfected with 483-3p-M or 483-3p-I. mut: mutant; M-CT: mimic control; 483-3p-M: mimic of miR-483-3p.

The biological process underlying the regulation of adipogenesis by miR-483-3p was further illustrated by the bioinformatics analysis of potential miR-483-3p target genes using the TargetScanHuman 7.0 and miRWalk online databases. Through database prediction, we selected *IGF1*, O-linked N-acetylglucosamine transferase (*OGT*), and chloride voltage-gated channel (*CLCN3*) as potential targets of miR-483-3p. We cloned the 3′ UTRs of each of these three genes separately into a dual-luciferase-based reporter plasmid and cloned miR-483-3p into pSUPER. The miR-483-3p plasmid or control plasmid was then co-transfected with one of the three reporter plasmids into HEK-293T cells. While the presence of miR-483-3p significantly decreased the luciferase reporter activity of *IGF1*, the decreases in the activities of *OGT* and *CLCN3* were not significant (Supplementary Figure 3). We also created a mutation of eight nucleotides within the mRNA binding site of miR-483-3p to interrupt the hypothetical connection between the *IGF1* mRNA and miR-483-3p (Figure 4A, 4B). As shown in Figure 4B, the mutant miR-483-3p restored the luciferase activity of *IGF1*.

HADSCs were also transfected with 483-3p-I or 483-3p-M (mimic of miR-483-3p), and RT-qPCR was used to quantify the mRNA levels of *IGF1*. *IGF1* expression was downregulated by miR-483-3p overexpression (Figure 4C) and upregulated by miR-483-3p inhibition (Figure 4D). These observations generally suggested that *IGF1* is a direct target gene of miR-483-3p.

### MiR-483-3p induces senescence of hADSCs through *IGF1*

**Figure 5:**
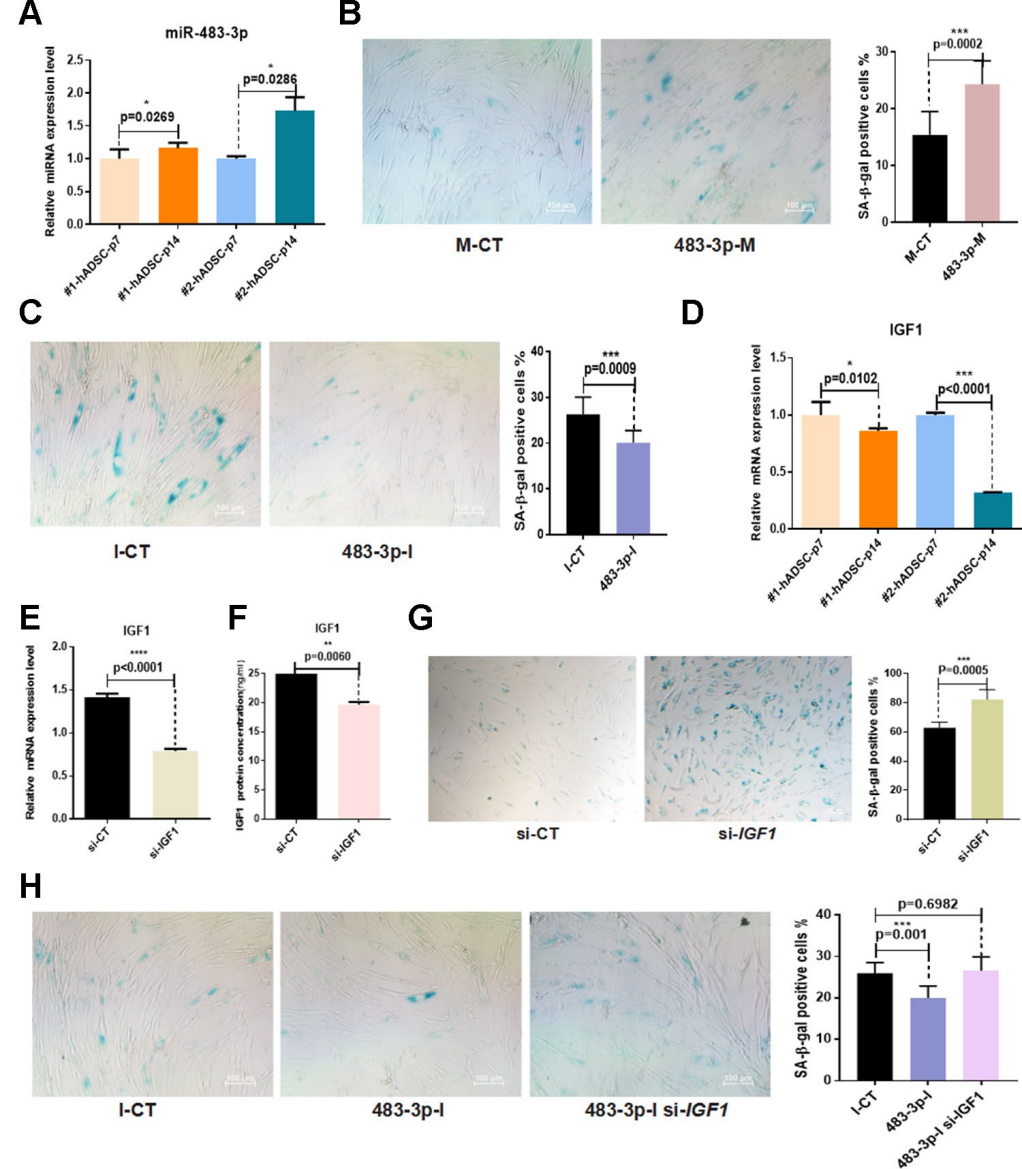
**Effects of miR-483-3p and *IGF1* on senescence of hADSCs.** (**A**) MiR-483-3p expression at early-passage (P7) and late-passage (P14) hADSCs was detected by RT-qPCR. (**B**) SA-β-gal staining of hADSCs (P14) transfected with 483-3p-M and corresponding control (10×; scale: 100μm). (**C**) SA-β-gal staining of hADSCs (P14) transfected with 483-3p-I and corresponding control (10×; scale: 100μm). (**D**) *IGF1* expression in hADSCs at P7 and P14 was detected by RT-qPCR. (**E**, **F**) Efficiency of IGF1 inhibition was assayed by RT-qPCR and ELISA. (**G**) SA-β-gal staining of hADSCs (P14) transfected with si-CT or si-IGF1 (4×; scale: 100μm). (**H**) SA-β-gal staining of hADSCs (P14) co-transfected with 483-3p-I and si-IGF1 (10×; scale: 100μm).

Given that miR-483-3p was upregulated during adipogenic differentiation and that it controlled the expression of adipogenic differentiation markers and phenotypes, we sought to determine whether it was involved in hADSC senescence in vitro. The expression levels of miR-483-3p during early and late passages of hADSCs taken from two different donor samples were examined using RT-qPCR. MiR-483-3p expression was significantly increased in senescent cells (Figure 5A). To further investigate the biological effect of miR-483-3p on hADSC senescence phenotypes, hADSCs were transfected with 483-3p-I or 483-3p-M to alter the *in vitro* expression levels of miR-483-3p, then stained for SA-β-gal. Transfection with 483-3p-M increased the activity of SA-β-gal while 483-3p-I decreased it compared with the corresponding control group (Figure 5B, 5C). Interestingly, we also found that *IGF1* expression was significantly reduced in senescent cells (Figure 5D).

Based on these results, we transfected hADSCs with the small interfering RNA (siRNA) si-*IGF1* to inhibit endogenous *IGF1* expression. The results of RT-qPCR and ELISA showed that si-*IGF1* effectively reduced *IGF1* expression (Figure 5E, 5F). Additionally, the SA-β-gal activity of the si-*IGF1* transfection group was significantly higher than that of the si-NC group (Figure 5G).

To further verify the relationship between miR-483-3p and *IGF1* in the *in vitro* aging of hADSCs, we co-transfected hADSCs with 483-3p-I and si-*IGF1*. The knockdown of miR-483-3p reduced SA-β-gal activity, but the additional transfection of si-*IGFI* restored this activity in hADSCs compared with the control group (Figure 5H).

## DISCUSSION

The high abundance of hADSCs makes them the ideal source of adult stem cells in clinical and regenerative medicine. However, long-term culture of mesenchymal stem cells (MSCs) results in altered cell morphology, slower proliferation rates, and disruption of the balance between osteogenesis and adipogenesis [4, 5]. In our study, MSCs undergoing long-term proliferation displayed a significant increase in senescence, as evidenced by their shortened telomere length and increased expression of SA-β-gal, p16, p21, and p53. These results concur with the findings of previous studies [22, 23].

While many studies have focused on the potential of aging MSCs to undergo adipogenic and osteogenic differentiation, several of these studies have reported conflicting findings. Most studies have reported that MSCs undergoing long-term culture exhibit a decline in all differentiation abilities, but the results of some studies have suggested the opposite [24, 25]. Lei et al. demonstrated that the osteogenic potential decreased during replicative senescence in MSCs, while the adipogenic potential increased in late passages [18]. Likewise, the results of our study indicated that the osteogenic differentiation potential decreases as the adipogenic differentiation potential increases in late passages. However, the mechanism of this phenomenon remains unknown.

Accumulating evidence suggests that miRNAs induce mRNA degradation and translational repression to regulate various cellular processes, such as proliferation, apoptosis, and differentiation [26]. Motoi et al. reported that miR-195 was highly expressed in old MSCs, which directly inactivated telomerase reverse transcriptase (TERT) and facilitated the senescence of MSCs, and abrogation of miR-195 could reverse stem cell senescence [27]. Another study by Wang et al. showed that miR-26b-3p was drastically upregulated during continuous *in vitro* passaging of human umbilical cord-derived MSCs, and played a vital role in regulating their proliferation [28].

Replicative senescence is a process marked by continuous, cumulative changes, and the resultant “aging phenotype” of hADSCs becomes more obvious with successive passaging. Accordingly, we chose late passage hADSCs to identify potential miRNAs that exert crucial functions during adipogenic differentiation and replicative senescence of hADSCs. MiR-483-3p expression was significantly upregulated during the adipogenic differentiation, and miR-483-3p inhibition limited the adipogenic potential of hADSCs. To elucidate the molecular mechanisms by which miR-483-3p regulates adipogenic differentiation, we performed the bioinformatics analysis of potential miR-483-3p target genes using the online databases. Notably, bioinformatics analysis and experimental data confirmed that *IGF1* is a direct target of miR-483-3p. In addition, there was a negative correlation between *IGF1* and miR-483-3p expression at the protein and mRNA levels in hADSCs.

IGF1*,* a primary hormone of growth and fat metabolism, is known to work through the binding of insulin-like growth factor receptor. The complex effects of IGF1 on normal growth and survival have been extensively studied, and while some research articles offer insightful observations, they also highlight paradoxes. The use of calorie restriction to inhibit IGF1 and mTOR has been widely demonstrated as an efficient anti-aging strategy because it was the most effective inducer of autophagy [29, 30]. However, an inactive form of *IGF1* results in severe growth retardation and proliferative defects in mice [31, 32]. IGF1 is considered to be the most potent growth factor for the prevention of apoptosis in eukaryotic cells [33]. Several prospective studies investigated the role of IGF1 in promoting PI3K/AKT signaling, which promotes cell proliferation and inhibits cell senescence [34–36]. IGF1 also regulates the proliferation and self-renewal of stem cells [37], and plays an essential role in skeletal development by promoting proliferation and osteogenic differentiation of MSCs [38].

Previous research has demonstrated that miR-483 is widely involved in a variety of cancers through the inhibition of proliferation, invasion and migration, and the induction of apoptosis [39–41]. Until this study, the effect of miR-483-3p on the replicative senescence of hADSCs remains unclear. We investigated that miR-483-3p was highly expressed, while *IGF1* mRNA was expressed at a low level in late-passage hADSCs. The knockdown of miR-483-3p inhibited the senescence of hADSCs, while simultaneous knockdown of *IGFI* restored the effect of miR-483-3p.

There are some drawbacks and limitations to our study. Firstly, miR-483 is located in intron 2 of *IGF2* and we found that miR-483 and *IGF2* have similar expression pattern in adipogenic differentiated hADSCs. However, our study only demonstrated the effects of miR-483-3p on hADSCs, further investigations of *IGF2* were needed. Secondly, although miR-483-3p was involved in replicative senescence of hADSCs, its exact role remains inconclusive. The questions of whether miR-483-3p plays a substantial role in aging and whether miR-483-3p inhibition can reverse the development of aging in vivo must be addressed in future studies.

In conclusion, we identified miR-483-3p as a key promoter of adipogenesis and a vital player in the regulation of replicative senescence in hADSCs through *IGF1* inhibition. MiR-483-3p also appears to be a novel indicator of replicative senescence in hADSCs. These findings provide essential clues for manipulating *IGF1* via miR-483-3p to attenuate aging and promote the proliferation of hADSCs.

## MATERIALS AND METHODS

### Isolation and characterization of hADSCs

Samples of subcutaneous adipose tissues were obtained from patients without malignant tumors, autoimmune diseases, congenital diseases, or genetic diseases, at the age of 30 (#1) and 34 (#2) undergoing fracture surgery. The operation protocols were approved by the Ethics Research Committee, Tongji University School of Medicine.

Tissue was excised from blood vessels and sliced into approximately 1 mm^3^ size, digested in a 0.2% collagenase type I/II mixture (Gibco, New York, USA) at 37°C for 1 hour, filtered with 70-μm nylon mesh and then subjected to centrifugation at 1,500 rpm for 10 minutes. The cells were cultured in DMEM/F12 medium supplemented with 10% fetal bovine serum, 1% non-essential amino acids, 100 U/mL penicillin and 100 μg/mL streptomycin, in a 5% CO2 environment at 37°C. After one week of culture when the cells reached about 80% confluence, they were passaged and transferred one-third of cells to a new culture dish (passage 1, P1). At P2, surface antigen sorting was performed on adherent and spindle-shaped hADSCs according to the following criteria: positive for CD73, CD90, and CD105 and negative for CD34, CD45, CD11b, CD19 and HLA-DR (BD, Human MSC Analysis Kit). Adipogenic and osteogenic differentiation kits (Gibco, New York, USA) were used to induce adipogenesis and osteogenesis, respectively. The adipocyte phenotype was evaluated using Oil Red O staining according to the previously described protocols [23]. Osteoblast phenotype was assessed using BCIP/NBT Alkaline Phosphatase staining assay kit according to the manufacturer’s protocols (Beyotime, Shanghai, China).

### Cell proliferation CCK-8 assay

Cell proliferation ability was evaluated using CCK-8 assay (Dojindo, Shanghai, China) in early-passage and late-passage hADSCs according to the manufacturer’s instructions. Briefly, cells were cultured in 96-well plates at a concentration of 1 × 10^4^ cells/well. After 24 hours of culture, 10 μL CCK-8 reagent was added to each well and further incubated for 4 hours. The absorbance was determined at a wavelength of 450nm using SpectraMax M5 (Molecular Devices, CA, USA).

### MiRNAs sequencing

The miRNAs samples were extracted using the mirVana™ miRNA Isolation Kit (Ambion, Waltham, USA) following the manufacturer's instructions. Small RNA libraries were prepared using the Small RNA Sample Preparation kit (Illumina, SanDiego, USA) according to the manufacturer’s protocols. The quality of the purified small RNA sequencing libraries was confirmed on an Agilent 2100 Bioanalyzer. The small RNA sequencing data were first cleaned using small RNA sequencing data cleaning pipeline. Next, the clean sequence data were aligned to miRBase to detect and estimate the expression of microRNAs. Subsequently, the remaining read sequences were aligned to the fRNAdb to get the expression of various non-coding RNAs. The miRNAs sequencing data generated in this study have been deposited in the NCBI Gene Expression Omnibus (GEO) under accession number GSE138879.

### Cell transfection

Mimics of miR-483-3p (483-3p-M) and miR-483-5p (483-5p-M), inhibitors of miR-483-3p (483-3p-I), and their corresponding negative controls (mimic-NC (M-CT) and inhibitor-NC (I-CT)), and the siRNA of *IGF1* (si-*IGF1*) and its corresponding negative control (si-NC) were synthesized by Genepharma (Shanghai, China). hADSCs were grown until 50%–60% confluence then transfected with 100 nM miRNA mimic, inhibitor or NC using Lipofectamine 2000 reagent (Invitrogen, CA, USA). The culture medium was changed after 12 hours of transfection and cellular differentiation was induced 48 hours later according to the manufacturer’s recommendations.

### Dual-luciferase reporter assay

A partial sequence of *IGF1* containing the predicted miR-483-3p binding site was amplified by PCR and cloned into the dual-luciferase plasmid. The miR-483-3p gene and a mutant ‘seed’ sequence were synthesized by Thermo Fisher Scientific and cloned into the pSUPER vector. Reporter assays were performed in HEK-293T cells, which were harvested 32 hours after transfection. Firefly and Renilla luciferase activities were measured using the Dual-Glo Luciferase assay system (Promega, Madison, USA). A signal from the green Renilla luciferase intracellular protein was normalized to the red firefly luciferase signal.

### RT-qPCR analysis

Total RNA was extracted using Trizol reagent (Invitrogen, California, USA). The quality and concentration of total RNA were determined using a nanodrop 2000 and separation on a 1% agarose gel. Reverse transcription and detection of miRNAs were accomplished using the all-in-one miRNA qRT-PCR detection kit (Genecopoeia, Maryland, USA) according to the manufacturer’s instructions. MRNA expression was quantified via an initial synthesis of cDNA using a PrimeScript RT reagent kit (Takara, Dalian, China), followed by RT-qPCR using SYBR Green SuperMix (Bio-Rad, California, USA) on the Quantstudio 7 Flex Real-Time PCR system (Thermo Scientific, MA, USA). Each sample was tested in triplicate. The relative expression levels of miRNAs and mRNA were normalized to the expression of U6 and *β-actin*, respectively. The relative gene expression was calculated using the comparative CT (2^-ΔΔCT^) method, and the sequences of the primers used were listed in Table 1.

**Table 1:** Primer sequences used in this study.

**Target gene**	**Forward primer (5'--3')**	**Reverse primer (5'--3')**
β-actin	F: ACCCACACTGTGCCCATCT	R: ATGTCACGCACGATTTCCC
p16	F: TTCCTGGACACGCTGGT	R: GGTTACTGCCTCTGGTGC
p21	F: TTAGCAGCGGAACAAGGA	R: AAGACAACTACTCCCAGCCC
p53	F: TGCATTTTCACCCCACCCTT	R: ACACAGGTGGCAGCAAAGTT
IGF1	F: ATGCTCTTCAGTTCGTGTGTGG	R:CAATACATCTCCAGCCTCCTTAGA
IGF2	F: GCTGGCAGAGGAGTGTCC	R: AGGTGAGAAGCACCAGCATC
TEL	F:GGTTTTTGAGGGTGAGGGTGAGGGTGAGGGTGAGGGT	R:TCCCGACTATCCCTATCCCTATCCCTATCCCTATCCCTA
36B4	F: CAGCAAGTGGGAAGGTGTAATCC	R:CCCATTCTATCATCAACGGGTACAA
LPL	F:CTGGACGGTAACAGGAATGTATGAG	R: CATCAGGAGAAAGACGACTCGG
PPARγ	F: CCTATTGACCCAGAAAGCGATT	R: CATTACGGAGAGATCCACGGA
RUNX2	F:TGTCATGGCGGGTAACGAT	R:AAGACGGTTATGGTCAAGGTGAA
miR-483-3p	F:TCACTCCTCTCCTCCCGTCTT	
miR-483-5p	F:AAGACGGGAGGAAAGAAGGGAG	
U6	F:GCTTCGGCAGCACATATACTAAAAT	

Note: F: forward; R: reverse; TEL: Telomere DNA sequence

### Western blot analysis

Cells were washed twice with cold phosphate-buffered saline, then lysed with RIPA lysis buffer containing protease inhibitor and shaken for 15 minutes on ice. Protein samples were subjected to centrifugation at 12,000 ×g for 15 minutes, and the supernatants were collected. Protein concentration was determined using bicinchoninic acid protein assay kit (Beyotime). Samples containing equal amounts of protein were separated by sodium dodecyl sulfate-polyacrylamide gel electrophoresis and then transferred onto a 0.22-μm polyvinylidene difluoride membrane. After blocking with 5% non-fat milk (Sigma, St. Louis, USA), the membranes were incubated with a primary antibody (PPARγ: ab191407, Abcam, Cambridge, UK; LPL: ab172953, Abcam; α-tubulin: T9026, Sigma) at 4°C overnight, then further incubated with the corresponding secondary antibody (170-6515 and 170-6516, Bio-Rad) according to the manufacturer’s instructions. Protein bands were quantified using the Amersham Imager system.

### Assay of cellular senescence (SA-β-gal staining)

Cells were cultured in 24-well plates at a concentration of 2 × 10^4^ cells/well. SA-β-gal activity was determined using the Cell Senescence β-Galactosidase Staining Kit (Beyotime) according to the manufacturer’s instructions. The nuclei were stained with DAPI, and blue-stained senescent cells were counted using Image J.

### ELISA

The protein content of IGF1 in cells was determined using the Human IGF1 enzyme-linked immunosorbent assay (ELISA) kit (Elabscience, Wuhan, China) according to the manufacturer’s instructions.

### Statistical analysis

Results are express as the means ± standard deviation (n=3). Student's t-test was used to determine significance between two groups. A P-value <0.05 was considered statistically significant.

## Supplementary Material

Supplementary Figures

## References

[1] Chang YH, Liu HW, Wu KC, Ding DC. Mesenchymal Stem Cells and Their Clinical Applications in Osteoarthritis. Cell transplantation. 2016; 25:937–950. 10.3727/096368915X690288 26688464

[2] Li N, Hua J. Interactions between mesenchymal stem cells and the immune system. Cell Mol Life Sci. 2017; 74:2345–60. 10.1007/s00018-017-2473-5 28214990 PMC11107583

[3] Wong SP, Rowley JE, Redpath AN, Tilman JD, Fellous TG, Johnson JR. Pericytes, mesenchymal stem cells and their contributions to tissue repair. Pharmacol Ther. 2015; 151:107–20. 10.1016/j.pharmthera.2015.03.006 25827580

[4] Wagner W, Bork S, Lepperdinger G, Joussen S, Ma N, Strunk D, Koch C. How to track cellular aging of mesenchymal stromal cells? Aging (Albany NY). 2010; 2:224–30. 10.18632/aging.100136 20453259 PMC2881510

[5] Baker N, Boyette LB, Tuan RS. Characterization of bone marrow-derived mesenchymal stem cells in aging. Bone. 2015; 70:37–47. 10.1016/j.bone.2014.10.014 25445445

[6] Turinetto V, Vitale E, Giachino C. Senescence in human mesenchymal stem cells: functional changes and implications in stem cell-based therapy. Int J Mol Sci. 2016; 17:1164. 10.3390/ijms17071164 27447618 PMC4964536

[7] Ambros V, Bartel B, Bartel DP, Burge CB, Carrington JC, Chen X, Dreyfuss G, Eddy SR, Griffiths-Jones S, Marshall M, Matzke M, Ruvkun G, Tuschl T. A uniform system for microRNA annotation. RNA. 2003; 9:277–79. 10.1261/rna.2183803 12592000 PMC1370393

[8] Bartel DP. MicroRNAs: genomics, biogenesis, mechanism, and function. Cell. 2004; 116:281–97. 10.1016/s0092-8674(04)00045-5 14744438

[9] Mohr AM, Mott JL. Overview of microRNA biology. Semin Liver Dis. 2015; 35:3–11. 10.1055/s-0034-1397344 25632930 PMC4797991

[10] Cullen BR. Transcription and processing of human microRNA precursors. Mol Cell. 2004; 16:861–65. 10.1016/j.molcel.2004.12.002 15610730

[11] Qadir MI, Faheem A. miRNA: a diagnostic and therapeutic tool for pancreatic cancer. Crit Rev Eukaryot Gene Expr. 2017; 27:197–204. 10.1615/CritRevEukaryotGeneExpr.2017019494 29199604

[12] Price NL, Ramírez CM, Fernández-Hernando C. Relevance of microRNA in metabolic diseases. Crit Rev Clin Lab Sci. 2014; 51:305–20. 10.3109/10408363.2014.937522 25034902

[13] Choi SW, Lee JY, Kang KS. miRNAs in stem cell aging and age-related disease. Mech Ageing Dev. 2017; 168:20–29. 10.1016/j.mad.2017.08.013 28847486

[14] Liu M, Roth A, Yu M, Morris R, Bersani F, Rivera MN, Lu J, Shioda T, Vasudevan S, Ramaswamy S, Maheswaran S, Diederichs S, Haber DA. The IGF2 intronic miR-483 selectively enhances transcription from IGF2 fetal promoters and enhances tumorigenesis. Genes Dev. 2013; 27:2543–48. 10.1101/gad.224170.113 24298054 PMC3861668

[15] Anderson BA, McAlinden A. miR-483 targets SMAD4 to suppress chondrogenic differentiation of human mesenchymal stem cells. J Orthop Res. 2017; 35:2369–77. 10.1002/jor.23552 28244607 PMC5573664

[16] Cawthon RM. Telomere measurement by quantitative PCR. Nucleic Acids Res. 2002; 30:e47. 10.1093/nar/30.10.e47 12000852 PMC115301

[17] Chen Q, Shou P, Zheng C, Jiang M, Cao G, Yang Q, Cao J, Xie N, Velletri T, Zhang X, Xu C, Zhang L, Yang H, et al. Fate decision of mesenchymal stem cells: adipocytes or osteoblasts? Cell Death Differ. 2016; 23:1128–39. 10.1038/cdd.2015.168 26868907 PMC4946886

[18] Lei Q, Liu T, Gao F, Xie H, Sun L, Zhao A, Ren W, Guo H, Zhang L, Wang H, Chen Z, Guo AY, Li Q. Microvesicles as potential biomarkers for the identification of senescence in human mesenchymal stem cells. Theranostics. 2017; 7:2673–89. 10.7150/thno.18915 28819455 PMC5558561

[19] Yang L, Shi CM, Chen L, Pang LX, Xu GF, Gu N, Zhu LJ, Guo XR, Ni YH, Ji CB. The biological effects of hsa-miR-1908 in human adipocytes. Mol Biol Rep. 2015; 42:927–35. 10.1007/s11033-014-3830-1 25421647

[20] Bergman D, Halje M, Nordin M, Engström W. Insulin-like growth factor 2 in development and disease: a mini-review. Gerontology. 2013; 59:240–49. 10.1159/000343995 23257688

[21] Diao S, Yang H, Cao Y, Yang D, Fan Z. IGF2 enhanced the osteo-/dentinogenic and neurogenic differentiation potentials of stem cells from apical papilla. J Oral Rehabil. 2019. [Epub ahead of print]. 10.1111/joor.12859 31291686

[22] Kim J, Kim Y, Choi H, Kwon A, Jekarl DW, Lee S, Jang W, Chae H, Kim JR, Kim JM, Kim M. Ubiquitin C decrement plays a pivotal role in replicative senescence of bone marrow mesenchymal stromal cells. Cell Death Dis. 2018; 9:139. 10.1038/s41419-017-0032-5 29382826 PMC5833785

[23] Park JS, Kim HY, Kim HW, Chae GN, Oh HT, Park JY, Shim H, Seo M, Shin EY, Kim EG, Park SC, Kwak SJ. Increased caveolin-1, a cause for the declined adipogenic potential of senescent human mesenchymal stem cells. Mech Ageing Dev. 2005; 126:551–59. 10.1016/j.mad.2004.11.014 15811424

[24] Bonab MM, Alimoghaddam K, Talebian F, Ghaffari SH, Ghavamzadeh A, Nikbin B. Aging of mesenchymal stem cell in vitro. BMC Cell Biol. 2006; 7:14. 10.1186/1471-2121-7-14 16529651 PMC1435883

[25] Wagner W, Horn P, Castoldi M, Diehlmann A, Bork S, Saffrich R, Benes V, Blake J, Pfister S, Eckstein V, Ho AD. Replicative senescence of mesenchymal stem cells: a continuous and organized process. PLoS One. 2008; 3:e2213. 10.1371/journal.pone.0002213 18493317 PMC2374903

[26] Tomé M, López-Romero P, Albo C, Sepúlveda JC, Fernández-Gutiérrez B, Dopazo A, Bernad A, González MA. miR-335 orchestrates cell proliferation, migration and differentiation in human mesenchymal stem cells. Cell Death Differ. 2011; 18:985–95. 10.1038/cdd.2010.167 21164520 PMC3131940

[27] Okada M, Kim HW, Matsu-ura K, Wang YG, Xu M, Ashraf M. Abrogation of age-induced MicroRNA-195 rejuvenates the senescent mesenchymal stem cells by reactivating telomerase. Stem Cells. 2016; 34:148–59. 10.1002/stem.2211 26390028 PMC4797648

[28] Wang Q, Xu C, Zhao Y, Xu Z, Zhang Y, Jiang J, Yan B, Gu D, Wu M, Wang Y, Liu H. miR-26b-3p regulates human umbilical cord-derived mesenchymal stem cell proliferation by targeting estrogen receptor. Stem Cells Dev. 2016; 25:415–26. 10.1089/scd.2015.0267 26723394

[29] Barzilai N, Huffman DM, Muzumdar RH, Bartke A. The critical role of metabolic pathways in aging. Diabetes. 2012; 61:1315–22. 10.2337/db11-1300 22618766 PMC3357299

[30] Johnson SC. Nutrient sensing, signaling and ageing: the role of IGF-1 and mTOR in ageing and age-related disease. Subcell Biochem. 2018; 90:49–97. 10.1007/978-981-13-2835-0_3 30779006

[31] Liu JP, Baker J, Perkins AS, Robertson EJ, Efstratiadis A. Mice carrying null mutations of the genes encoding insulin-like growth factor I (Igf-1) and type 1 IGF receptor (Igf1r). Cell. 1993; 75:59–72. 8402901

[32] Powell-Braxton L, Hollingshead P, Warburton C, Dowd M, Pitts-Meek S, Dalton D, Gillett N, Stewart TA. IGF-I is required for normal embryonic growth in mice. Genes Dev. 1993; 7:2609–17. 10.1101/gad.7.12b.2609 8276243

[33] Bianchi VE, Locatelli V, Rizzi L. Neurotrophic and neuroregenerative effects of GH/IGF1. Int J Mol Sci. 2017; 18:2441. 10.3390/ijms18112441 29149058 PMC5713408

[34] Ma X, Xu W, Zhang Z, Liu N, Yang J, Wang M, Wang Y. Salvianolic acid B ameliorates cognitive deficits through IGF-1/Akt pathway in rats with vascular dementia. Cell Physiol Biochem. 2017; 43:1381–91. 10.1159/000481849 28992623

[35] Zhou W, Wang J, Qi Q, Feng Z, Huang B, Chen A, Zhang D, Li W, Zhang Q, Bjerkvig R, Li X, Wang J. Matrine induces senescence of human glioblastoma cells through suppression of the IGF1/PI3K/AKT/p27 signaling pathway. Cancer Med. 2018; 7:4729–43. 10.1002/cam4.1720 30079478 PMC6143938

[36] Luo X, Jiang X, Li J, Bai Y, Li Z, Wei P, Sun S, Liang Y, Han S, Li X, Zhang B. Insulin-like growth factor-1 attenuates oxidative stress-induced hepatocyte premature senescence in liver fibrogenesis via regulating nuclear p53-progerin interaction. Cell Death Dis. 2019; 10:451. 10.1038/s41419-019-1670-6 31171766 PMC6554350

[37] Bendall SC, Stewart MH, Menendez P, George D, Vijayaragavan K, Werbowetski-Ogilvie T, Ramos-Mejia V, Rouleau A, Yang J, Bossé M, Lajoie G, Bhatia M. IGF and FGF cooperatively establish the regulatory stem cell niche of pluripotent human cells in vitro. Nature. 2007; 448:1015–21. 10.1038/nature06027 17625568

[38] Chen CY, Tseng KY, Lai YL, Chen YS, Lin FH, Lin S. Overexpression of insulin-like growth factor 1 enhanced the osteogenic capability of aging bone marrow mesenchymal stem cells. Theranostics. 2017; 7:1598–611. 10.7150/thno.16637 28529639 PMC5436515

[39] Yu FY, Zhou CY, Liu YB, Wang B, Mao L, Li Y. miR-483 is down-regulated in gastric cancer and suppresses cell proliferation, invasion and protein O-GlcNAcylation by targeting OGT. Neoplasma. 2018; 65:406–14. 10.4149/neo_2018_170608N411 29788742

[40] Niu ZY, Li WL, Jiang DL, Li YS, Xie XJ. Mir-483 inhibits colon cancer cell proliferation and migration by targeting TRAF1. Kaohsiung J Med Sci. 2018; 34:479–86. 10.1016/j.kjms.2018.04.005 30173777 PMC11915647

[41] Xiang Y, Song Y, Li Y, Zhao D, Ma L, Tan L. miR-483 is down-regulated in polycystic ovarian syndrome and inhibits KGN cell proliferation via targeting insulin-like growth factor 1 (IGF1). Med Sci Monit. 2016; 22:3383–93. 10.12659/msm.897301 27662007 PMC5040236

