# Supplementary material for: MiR-483 induces senescence of human adipose-derived mesenchymal stem cells through IGF1 inhibition
Source: Aging (Albany NY). 2020 Aug 15;12(15):15756–70. doi: 10.18632/aging.103818 (PMC7467354; doi:10.18632/aging.103818)
Supplement: Supplementary Figures [file aging-12-103818-s001..pdf]

SUPPLEMENTARY FIGURES

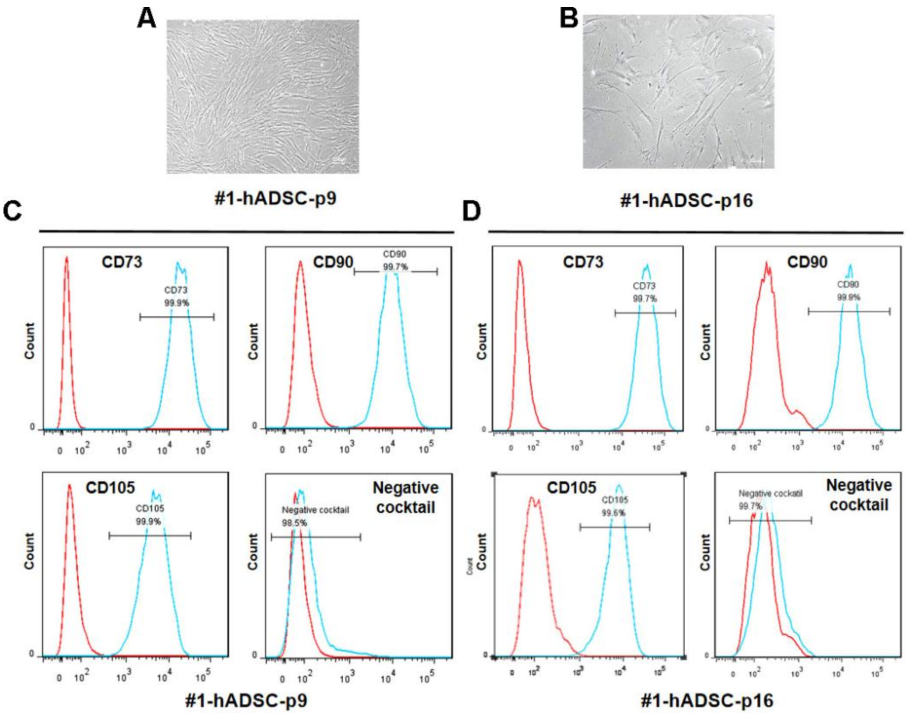

Supplementary Figure 1. Senescence phenotype of hADSCs at different passages. (**A, B**) The morphology of donor #1 hADSCs was observed under a microscope at P9 and P16. (**C, D**) Immunophenotypic characterization of #1hADSCs at P9 and P16 by flow cytometry.

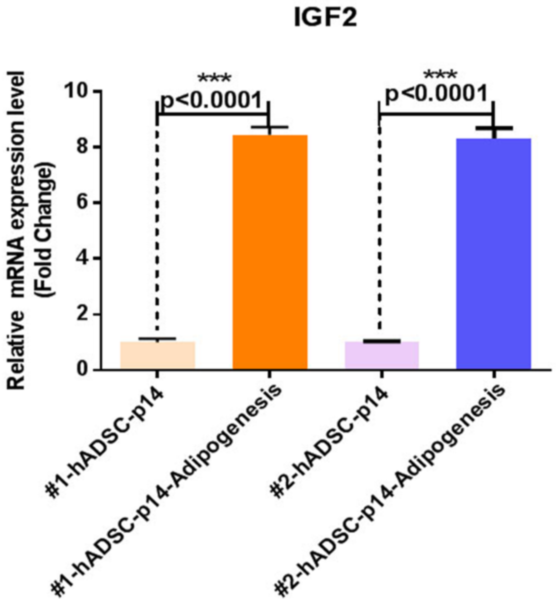

Supplementary Figure 2. *IGF2* expression during adipogenic differentiation of hADSCs was analyzed by RT-qPCR.

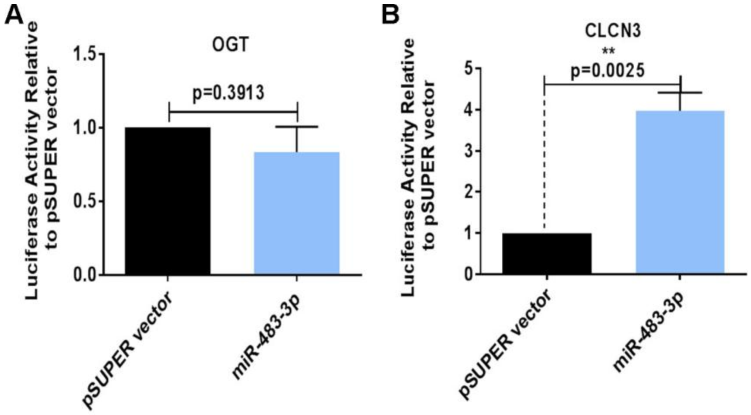

Supplementary Figure 3. Luciferase reporter assay analysis of miR-483 potential target genes. (**A**, **B**) Luciferase reporter activity in HEK-293T cells co-transfected with the luciferase reporter plasmid containing *OGT* and *CLCN3* 3′UTRs and wild-type pSUPER-miR-483-3p or empty pSUPER vector. Luciferase activity was assayed 32 hours after transfection.
